# Emergency department diagnosis of infective endocarditis using bedside emergency ultrasound

**DOI:** 10.1186/2036-7902-5-1

**Published:** 2013-02-11

**Authors:** Dina Seif, Andrew Meeks, Thomas Mailhot, Phillips Perera

**Affiliations:** 1 Department of Emergency Medicine, Los Angeles County + USC Medical Center, 1200 N. State St #1011, Los Angeles, CA, 90033, USA; 2 Divison of Emergency Medicine, Stanford University Medical Center, 300 Pasteur Drive, Alway Building, M121, Stanford, CA, 94305, USA

**Keywords:** Emergency ultrasound, Infective endocarditis, Transthoracic echocardiography, Cardiac valvular vegetation, Cardiac valvular regurgitation, Cardiac shunt, Septic emboli

## Abstract

Infective endocarditis is a challenging diagnosis that is rarely made in the emergency department. As the use of focused emergency ultrasound expands into more applications, including advanced echocardiography, the diagnosis of infective endocarditis may be made earlier, potentially leading to more timely treatment. We report a case of an ill-appearing patient presenting to the emergency department with an indwelling central venous catheter, a cardiac murmur, and necrotic toes, who was diagnosed with a large tricuspid vegetation and prominent tricuspid regurgitation on bedside emergency ultrasound. A cardiologist-performed echocardiogram confirmed these findings during the patient's hospital admission.

## Background

Infective endocarditis (IE) is a serious disease that requires prompt recognition and early treatment to minimize morbidity and mortality. However, it remains an elusive diagnosis for the emergency physician (EP) due to its protean manifestations. Fever, murmur, and peripheral emboli are the characteristic clinical findings, but these clues may not be present in some patients or early in the course of the illness. The Duke criteria are a widely used collection of clinical and microbiologic findings that emphasize echocardiography and blood cultures as major criteria in the diagnosis of IE [1]. Prolonged hospitalization is usually necessary to collect the clinical data necessary to fulfill the Duke criteria. Transthoracic echocardiography (TTE) is often the initial imaging study performed to evaluate IE, followed by transesophageal echocardiography (TEE) if needed. While echocardiography is traditionally performed in the cardiology laboratory as part of the inpatient evaluation, bedside emergency ultrasound (EUS) may allow for earlier diagnosis in the emergency department (ED) by demonstrating cardiac valvular vegetations. Published reports indicate that vegetations as small as 6 mm may be seen on TTE [2]. Valvular incompetence in the setting of a vegetation is both a diagnostic and prognostic tool and can be diagnosed by color flow Doppler imaging. Positive findings suggestive of IE on bedside EUS can enable the EP to more rapidly initiate appropriate antibiotics and to obtain prompt consultation for a diagnosis that is not often made in the ED. In this case report, we describe how bedside EUS expedited the diagnosis, disposition, and treatment of a patient with confirmed IE.

## Case presentation

### Initial presentation

A 60 year-old male with a history of stage IIB rectal cancer was brought to the ED by his son for 2 weeks of progressive shortness of breath. The patient had previously undergone surgery and radiation for this disease. In addition, he had an indwelling central venous catheter with a portal reservoir for chemotherapy, which had not been used for the last year. The patient had recently stopped taking all of his medications and was refusing other care from his family.

On physical examination, the patient was alert and oriented to name and place, but was ill-appearing and had difficulty answering most questions. His initial vital signs were as follows: temperature 97.2°F, heart rate 41 beats per minute, blood pressure 84/53 mmHg, respiratory rate 38 breaths per minute, and oxygen saturation 82% on room air. An indwelling catheter was present in the right chest wall with the portal reservoir removed. The outer portion of the catheter appeared to have been poorly cared for, but there were no overt signs of infection. Pulmonary examination demonstrated rales bilaterally. Cardiac auscultation was significant for a 3/6 pan-systolic murmur that was best heard at the left sternal border. Further examination demonstrated large confluent purple macules on his lower extremities and black-colored toes [see Additional file 1: Image 1]. Peripheral pulses were 1+ and equal bilaterally.

The serum white blood cell count was 26,000/mm^3^ with a profound neutrophilia and 10 to 24 bands/100 WBC. The hemoglobin was 6.9 g/dL, platelets were 17,000/mm^3^, and lactate was 7.4 mmol/L. The chemistry panel was significant for bicarbonate of 17 mmol/L, creatinine of 1.55 mg/dL, and blood urea nitrogen of 68 mg/dL. Urinalysis revealed greater than 50 red blood cells per high power field without white blood cells, bacteria, or casts. Chest radiography demonstrated multiple bilateral mid-lung opacities. The central venous catheter tip was seen in the proximal superior vena cava.

A bedside cardiac EUS was performed by the EP. Using the subxiphoid cardiac window, a large vegetation was visualized on the tricuspid valve. In addition, the right atrium appeared enlarged [see Additional file 2: Image 2 and Additional file 3: Video 1]. Using color flow Doppler imaging, regurgitation was noted across the tricuspid valve [see Additional file 4: Image 3 and Additional file 5: Video 2]. A view taken from the apical four-chamber approach provided an additional view of the tricuspid vegetation [see Additional file 6: Image 4 and Additional file 7: Video 3].

A computed tomography (CT) scan of the chest revealed multiple areas of pulmonary consolidation and cavitary lesions, suggestive of septic emboli. Interestingly, the CT scan also noted a large filling defect near the tricuspid valve suggesting a large vegetation [see Additional file 8: Image 5]. It was presumed in the ED that the lesions to the patient's lungs and legs were the result of septic emboli from the heart. A patent foramen ovale, allowing a right-sided cardiac lesion to reach the left-sided systemic circulation, was hypothesized as the cause of these emboli. A comprehensive echocardiography study performed by the cardiology service was requested to evaluate these findings.

### ED course

Blood cultures were drawn immediately, and the patient was started on broad-coverage antibiotics. The indwelling central venous catheter was removed. After aggressive fluid resuscitation, the patient's hemodynamic status improved significantly. The cardiology and cardiothoracic surgery services were consulted emergently based on the bedside EUS findings.

### Hospital course

The patient was admitted to the intensive care unit where TEE confirmed multiple bulky vegetations attached to the tricuspid valve and protruding into the right atrium. Significant tricuspid regurgitation and resultant right atrial dilation were also found. Of note, a bubble study showed a patent foramen ovale, explaining the path of systemic emboli from the right-sided cardiac lesion to the lower extremities. Due to the presence of multi-organ failure and overwhelming sepsis, the patient was deemed a poor surgical candidate. Cardiac surgery was delayed until he could be stabilized. Unfortunately, the patient's clinical status continued to decline, and he developed respiratory distress and increasing altered mental status. The patient's family requested a ‘do not resuscitate, do not intubate’ order with no escalation of care. Despite continued supportive medical care, the patient died shortly thereafter, approximately 2 weeks after his admission from the ED.

## Conclusions

This case describes a complicated presentation of right-sided IE. The patient had multiple findings suggesting the diagnosis of IE including a new systolic murmur, cutaneous manifestations, and pulmonary infarcts. Although the patient had no prior history of cardiac valvular disease, the poorly maintained central venous catheter was a strong predisposing source for cardiac infection. An additional finding of microscopic hematuria, noted on ED evaluation, may have been a sign of renal infarct or glomerulonephritis, both of which are strongly associated with IE. Approximately 24 hours after the patient presented to the ED, blood cultures were reported positive for oxacillin-sensitive staphylococcus. Interestingly, without this microbiologic data, the patient did not meet criteria for the diagnosis of IE using the traditional Duke criteria. However, bedside EUS, performed as part of the initial patient assessment, immediately suggested the correct diagnosis and allowed for earlier initiation of appropriate therapy and consultation with the appropriate specialists.

A large multicenter prospective cohort study of patients with IE found in-hospital mortality rates of 18% [3]. Due to the high mortality risk, early recognition and diagnosis are essential for optimal treatment of IE. Given that TTE is non-invasive, has no major safety issues, and can be obtained easily, it is the current standard initial imaging test for the evaluation of IE. In comparison, TEE is an invasive test with a low, but defined, risk of complications. In addition, TEE can be extremely challenging to obtain in the ED. For these reasons, most clinicians will begin the evaluation of IE with a TTE, even with the knowledge that this test is less sensitive than TEE for the diagnosis of IE [4]. Studies have suggested that probe distance and scanning through the lung are responsible for the relatively poorer valvular imaging by TTE. Vegetation size may also affect TTE sensitivity. Directly comparing TTE and TEE, Erbel et al. found only 25% of vegetations <5 mm, and 70% of those between 6 to 10 mm were identified by TTE [2]. With advances in ultrasound technology, image quality has improved vastly in the years since the results of this 1988 study. A more recent study reported TTE sensitivity of 84% for vegetations larger than 10 mm [5]. However, in most cases, if the clinical risk for IE is high and the TTE is non-confirmatory, a TEE will be performed as part of the comprehensive evaluation due to its higher sensitivity and ability to detect smaller lesions.

Multiple studies have shown that vegetation size is directly correlated with complications and mortality. Sanfilippo et al. found that the probability of sustaining a complication of IE was 10% when vegetations were ≤6 mm in size, 50% for lesions 7 to 11 mm, and almost 100% for lesions >15 mm in size [6]. After adjusting for multiple factors, Nunes et al. demonstrated that vegetation length was the only independent predictor of in-hospital mortality [7]. Thuny et al. demonstrated that large vegetations (>10 mm) and/or high vegetation mobility were associated with an increased embolic risk [8]. Because larger IE lesions may be most amenable to detection on bedside EUS, focused emergency ultrasound may be helpful in identifying those patients who are at greatest risk from this disease. Armed with this knowledge, the detection of a large cardiac vegetation on bedside TTE by the EP in a patient with suspicion for IE can prompt earlier aggressive treatment and specialty consultation without undue delay.

This case report demonstrates the potential utility of bedside cardiac TTE using modern ultrasound systems as an initial imaging test in the ED for patients in whom the clinical suspicion for IE is high. Established primary goals of bedside EUS in the ED are the detection of pericardial effusions and the assessment of cardiac contractility, although this list is now expanding [9]. Advanced echocardiography techniques are being integrated into a rapidly growing number of resuscitation ultrasound protocols [10]. While assessment of cardiac valvular lesions must still be considered an advanced use of ultrasound that requires additional training, many EPs are performing echocardiography exams as part of their clinical evaluations. As EPs gather experience with this modality, abnormalities may be identified, and EPs performing bedside EUS should learn to recognize normal cardiac anatomy as well as pathologic cardiac findings.

In this case, the EP performing the ultrasound exam rapidly detected the presence of a tricuspid valve with an obviously abnormal finding. Because of the large size of the right atrium in relation to the right ventricle, tricuspid valvular incompetence was suspected. Color flow Doppler imaging was then used to evaluate the valve and confirmed the presence of tricuspid regurgitation. The presence of a large valvular vegetation and the associated tricuspid regurgitation gave the patient a more ominous prognosis and prompted more aggressive ED resuscitation and treatment. While EPs with additional ultrasound training may decide to initiate therapy and obtain consultations more rapidly based on their bedside EUS, it is generally advisable to obtain confirmatory testing as a routine part of the patient's treatment course. Furthermore, as TTE may not detect all cases of IE, if the clinical suspicion remains high, hospital admission for blood culture testing and comprehensive echocardiography (including possible TEE) remains the prudent course.

## Consent

Written informed consent was obtained from the patient for publication of this case report and any accompanying images. A copy of the written consent is available for review by the Editor-in-Chief of this journal.

## Competing interests

Dina Seif is a consultant and educational speaker for SonoSite, Inc. Phillips Perera is an educational consultant for SonoSite, Inc. The other authors have no competing interests to declare.

## Authors’ contributions

AM and PP were actively involved in the patient’s clinical care. AM drafted the ‘Case Presentation’ section of the manuscript. DS performed the literature review and drafted the manuscript. PP acquired and prepared the ultrasound images and videos. TM, PP, and DS revised the manuscript. All authors read and approved the final manuscript.

## Authors’ information

DS, TM, and PP are medical doctors with Registered Diagnostic Medical Sonographer (RDMS) credentials.

## Supplementary Material

Additional file 1: Image 1 Photograph of patient's legs and feet. Click here for file

Additional file 2: Image 2 Subxiphoid cardiac view with right atrial dilation and tricuspid vegetation. Click here for file

Additional file 3: Video 1 Subxiphoid cardiac view with right atrial dilation and tricuspid vegetation. Click here for file

Additional file 4: Image 3 Color flow Doppler demonstrating regurgitation across the tricuspid valve. Click here for file

Additional file 5: Video 2 Color flow Doppler demonstrating regurgitation across the tricuspid valve. Click here for file

Additional file 6: Image 4 Apical four-chamber view. Click here for file

Additional file 7: Video 3 Apical four-chamber view. Click here for file

Additional file 8: Image 5 Ungated CT image with large filling defect representing tricuspid vegetation. Click here for file
