# Supplementary figures and images for: Emergency department diagnosis of infective endocarditis using bedside emergency ultrasound
Source: Crit Ultrasound J. 2013 Feb 11;5(1):1. doi: 10.1186/2036-7902-5-1 (PMC3740785; doi:10.1186/2036-7902-5-1)

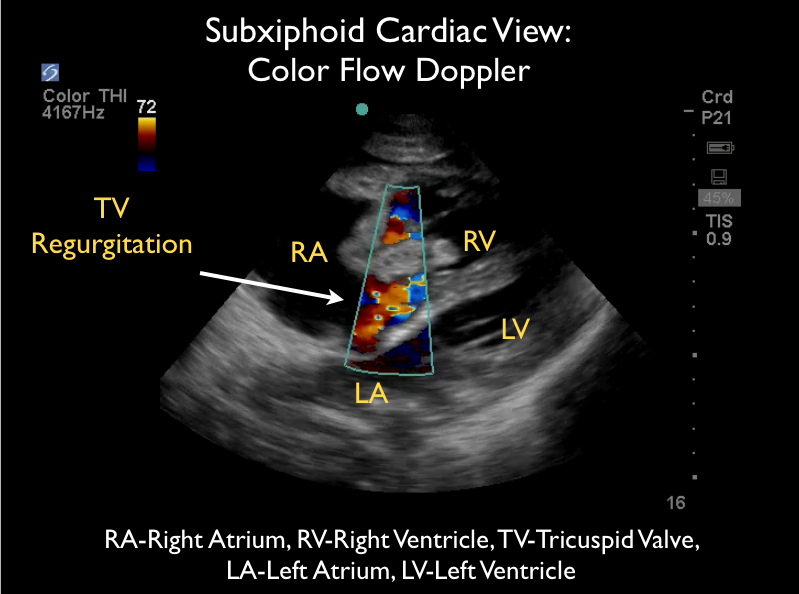

Supplement: Additional file 4: Image 3 — Color flow Doppler demonstrating regurgitation across the tricuspid valve. [file 2036-7902-5-1-S4.tiff]

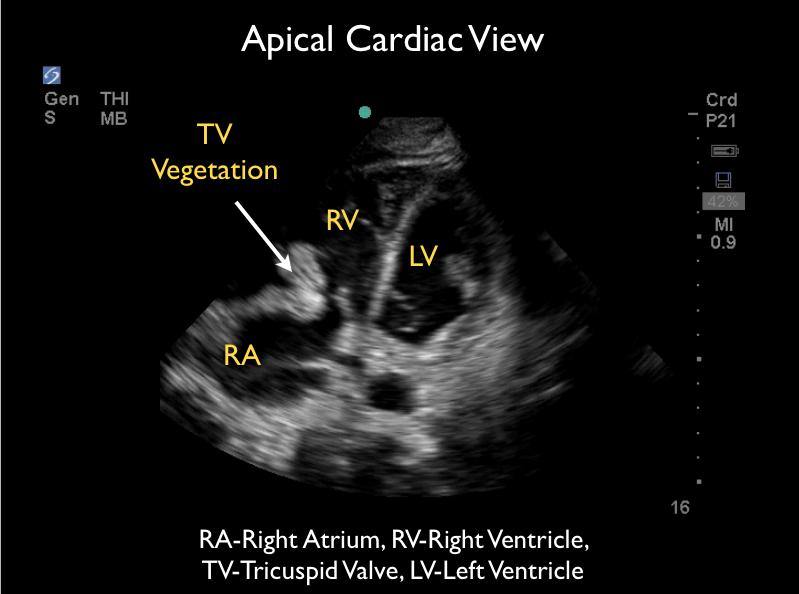

Supplement: Additional file 6: Image 4 — Apical four-chamber view. [file 2036-7902-5-1-S6.tiff]

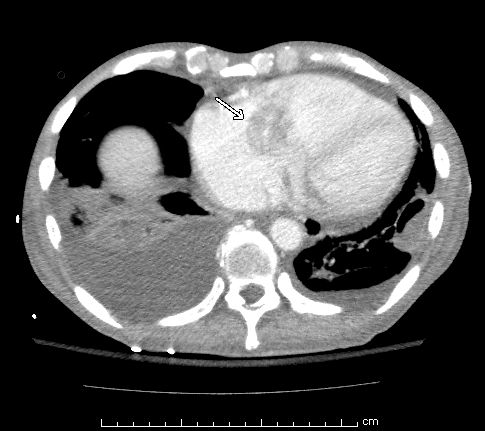

Supplement: Additional file 8: Image 5 — Ungated CT image with large filling defect representing tricuspid vegetation. [file 2036-7902-5-1-S8.jpeg]
